# Sugar-Based Eutectic Systems Combined with Cyclodextrins for Enhanced Solubility of Carvedilol

**DOI:** 10.3390/molecules31091490

**Published:** 2026-04-29

**Authors:** Alejandra Polo, Álvaro Werner, Estefanía Zuriaga, Beatriz Giner, Laura Lomba

**Affiliations:** Facultad de Ciencias de la Salud, Universidad San Jorge, Campus Universitario, Autov. A23 km 299, 50830 Villanueva de Gállego, Zaragoza, Spain; apolo@usj.es (A.P.); awerner@usj.es (Á.W.); ezuriaga@usj.es (E.Z.); bginer@usj.es (B.G.)

**Keywords:** cyclodextrins, natural deep eutectic solvents, carvedilol, solubility, drug

## Abstract

(1) Background: Carvedilol is a poorly water-soluble drug, which limits its therapeutic performance. Deep eutectic solvents (DES) and cyclodextrins (CD) are emerging solubilizing agents that can improve drug bioavailability. (2) Methods: Twenty-one DES were prepared using choline chloride and polyols or sugars (xylitol, sorbitol, glucose, and fructose) at different molar ratios with water. α and β cyclodextrins (CD) were added (0.5–2 mM) using two incorporation strategies: (Method 1) addition to the aqueous phase before DES formation; (Method 2) direct addition to the preformed DES. (3) Results: Carvedilol solubility markedly increased with DES–CD combinations. In Method 1, xylitol-based DES provided up to a 16-fold enhancement, especially with β-CD at low concentrations, while glucose and sorbitol systems showed modest effects. Fructose-based mixtures improved mainly at a 2:1:35 ratio without CDs. In Method 2, α-CD with xylitol or sorbitol yielded the highest increases (up to 38.9-fold). (4) Conclusions: The solubilization efficiency depends on DES composition, CD type, and concentration. α-CD combined with xylitol-based DES showed the best results, highlighting this approach as a promising strategy to enhance carvedilol solubility for pharmaceutical applications.

## 1. Introduction

**Figure 1:**
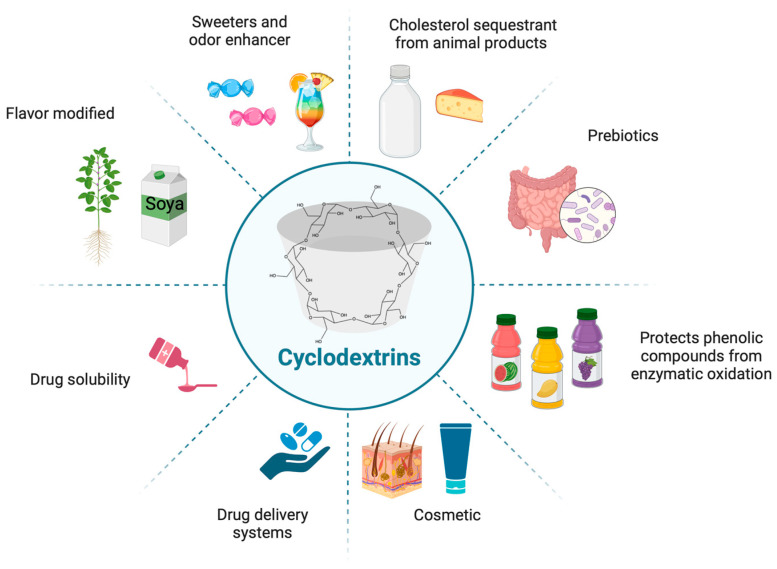
Food and health applications of cyclodextrins. Created with https://BioRender.com.

Cyclodextrins (CDs) are cyclic molecules made up of glucopyranose units arranged in a ring structure. Naturally, they are classified into three primary types, α, β and γ, containing 6, 7, and 8 glucose units, respectively. These variations in the number of glucose units lead to differences in their solubility and size. Structurally, CDs have a conical shape, characterized by a wider and narrower end, which arises from the spatial configuration of their glucopyranose units [1]. The structure of CDs features a hydrophilic outer surface, largely attributed to the hydroxyl groups, and a relatively hydrophobic internal cavity. This unique arrangement enables CDs to encapsulate various substances, modifying the solubility and thus enhancing their bioavailability and biocompatibility [2,3,4,5]. For these reasons, CDs have been employed in a number of applications [6] (Figure 1). For instance, in the food industry, cyclodextrins enhance organoleptic properties; their use in juices protect phenolic compounds from enzymatic oxidation, thereby improving the appearance of the product. Additionally, CDs have been shown to be helpful retaining aromas in various foods, thanks to the encapsulation of these volatile substances. Another interesting application is the use of CDs as sequesters and removal agents of harmful substances, such as cholesterol, from certain foods [7,8].

In the cosmetic industry, CDs can effectively stabilize compounds, preserving fragrances and flavors. They also enhance formulation performance by reducing vapor pressure, modifying water solubility, and improving the thermal stability of oils [9]. For these reasons, CDs are used in a variety of products such as toothpaste, creams, wipes, tissues, etc. [10,11]. In the healthcare field, CDs have demonstrated a wide range of applications. For example, they are used as drug carriers in cancer therapies, taking advantage of their ability to enhance drug permeation and retention within tumor cells [12,13]. Due to their ability to interact with cellular membranes, CDs are applicable to a wide range of substances, including oligosaccharides, proteins, and oligonucleotides. Furthermore, CDs can improve the delivery of plasmids, viral vectors, and antisense constructs [14] or can even be used as a prebiotic [15]. Cyclodextrins have also been utilized to enhance the solubility and stability of pharmaceuticals, making them valuable for the formulation of various active ingredients. Notably, several approved drugs incorporate cyclodextrins in their formulations, including tablets, creams, suppositories, capsules, and more [16]. Regarding the improvement of drug solubility, other techniques are traditionally used, such as the use of micronization [17,18] cosolvents, pH modification, salt formation, solid dispersion, complexation [19], hydrotropy [20], nanosuspension [21], and cocrystallization [22,23], among others. Finally, the use of deep eutectic solvents (DES) is currently being tested. These solvents consist of systems made up of two or more solid compounds at room temperature, which, through physical processing, experience a reduction in their melting point and transition into a liquid state [24,25].

DES are composed of a hydrogen bond acceptor (HBA) and a hydrogen bond donor (HBD) [26]. When natural components, typically plant primary metabolites like sugars, are used to synthesize DES, they are referred to as Natural Deep Eutectic Solvents (NADES) [27]. Various substances can function as HBAs, with the most common being quaternary salts like choline chloride or betaine. However, other compounds, such as thymol, menthol, or decanoic and lauric acids, can also play this role [28]. For HBD, several chemicals such as urea, amino acids, alcohols, amides, amines or carboxylic acids can be used [29].

In this study, a combination of cyclodextrins and deep eutectic systems was used to investigate the solubility of the active ingredient, carvedilol (1-(9H-Carbazol-4-yloxy)-3-[[2-(2-methoxyphenoxy)ethyl]-amino]-2-propanol, which is classified as a class II drug in the Biopharmaceutical Classification System (BCS). This means that carvedilol presents high permeability but low solubility. It is available in standard and extended-release tablets. However, it is typically formulated in tablet form (both standard and extended release). Nevertheless, oral solutions should be developed to improve patient adherence and bioavailability. Carvedilol is a nonselective beta-blocker with α1-adrenergic receptor antagonist properties [30,31] and a non-nonselective cardiac beta-blocker with peripheral vasodilating effects [32]. It is usually used in the chronic treatment of patients with hypertension, angina pectoris or even heart failure [33,34,35,36]. Furthermore, it has been clinically observed that this drug can decrease the morbidity and mortality in patients with heart failure [37,38,39]. The limited aqueous solubility of carvedilol is well established and has a direct impact on its pharmaceutical development, as evidenced by the fact that currently available formulations are restricted to oral solid dosage forms. Consequently, in hospital settings, carvedilol tablets are often crushed and combined with excipients to prepare extemporaneous liquid suspensions prior to administration. This practice introduces variability in dose accuracy, which can lead to underdosing or overdosing of the active pharmaceutical ingredient, thereby posing safety concerns for patients. In this context, the development of liquid formulations of carvedilol would offer significant clinical advantages, particularly for pediatric patients and individuals with dysphagia. Such formulations allow for more precise dose adjustments based on body weight and age. They can facilitate administration without the need to swallow solid dosage forms and reduce the risk of aspiration, ultimately enhancing treatment adherence and acceptability in vulnerable populations. Recent advances in the development of pediatric oral liquid formulations of carvedilol have demonstrated adequate stability and clinical applicability in pediatric cardiology, supporting these considerations. Furthermore, current regulatory guidelines emphasize the importance of age-appropriate formulations and prioritizing liquid dosage forms to optimize pharmacotherapy in these patient groups [40,41].

For all these reasons there have been various strategies tested by different authors to improve the aqueous solubility of carvedilol before. For example, Loftsson et al. conducted complexation studies with γ-cyclodextrin and HP-β-CD, which demonstrated increases in solubility of up to 10-fold through the formation of stable inclusion complexes [42]. Shewale et al. demonstrated that hydroxypropyl-β-cyclodextrin increases the apparent solubility of carvedilol in aqueous media, with favorable stability constants [43]. Zoghbi developed solid dispersions with PVP K-30 and Gelucire^®^ 50/13 that improved the dissolution rate by up to 50-fold [44]. Chiclana-Rodríguez et al. evaluated the solubility and stability of carvedilol in media of different pH levels. To this end, they used malic acid and studied its physical, chemical and microbiological stability in a 1 mg/mL solution. Stability was observed over a 12-month period and the use of methylparaben was not necessary [40]. Furthermore, our group previously used deep eutectic systems (DES) based on choline and glycerol, achieving 20- to 100-fold increases, although with challenges regarding long-term stability [45].

The eutectic mixtures used in this work have been formed by choline chloride and different sugars (xylitol, sorbitol, glucose and fructose) at different molar proportions with water in order to prepare a total of 21 moieties. Xylitol, sorbitol, glucose and fructose were selected as they are widely used pharmaceutical excipients that are biocompatible and rich in hydroxyl groups, making them suitable for the formation of aqueous eutectic systems and for modulating the solubility of poorly soluble drugs such as carvedilol [46,47,48]. In addition, these mixtures have been combined with α and β-cyclodextrins (α-CD and β-CD, respectively) at different molar concentrations (0.5, 1 and 2 mM) in order to overcome the limitations of carvedilol’s low aqueous solubility and improve its therapeutic effectiveness. These two cyclodextrins have been selected due to their different cavity sizes, which can influence the inclusion efficiency depending on the molecular dimensions of carvedilol. This comparison allows for the evaluation of how cavity size and host–guest compatibility affect the solubilization performance in the studied systems.

## 2. Results

### 2.1. Preparation of DES

Various methods of preparing DES have been reported in the literature, including evaporation, heating and freeze-drying. For this study, the heating method was chosen due to its simplicity and widespread use. The molar mass of each mixture was determined using the following equation:

MW_DES_ = X_choline chloride_·MW_choline chloride_ + X_sugar_·MW_sugar_ + X_water_·MW_water_ (1)

where X is the mole fraction and MW is the molar mass (the water term is added only if needed).

Table 1 presents the studied mixtures, the molecular mass and density at 25 °C as well as their physical appearance. For all the analyzed systems, the density is observed to decrease as the water content in the system increases. The densest systems are 2:1:4 for xylitol and 2:1:10 for the rest of the sorbitol, glucose and fructose systems.

A revision of the rheological behavior of these eutectic mixtures shows that yield shear stress decreases with increasing water content, except for glucose-based DES, for which values are minimal [24]. Xylitol-based DES exhibits the highest yield stress, followed by fructose, sorbitol, and glucose. The flow index increases with water content, indicating a shift toward Newtonian-like behavior, particularly at high water content. The consistency factor, representing viscosity, decreases with more water, aligning with the Herschel–Bulkley model and highlighting reduced viscosity and shear-thinning behavior in high-water-content mixtures. These authors previously published this information about these systems [24].

### 2.2. Calibration Curves

Table 2 compiles the results obtained for the calibration curves. The concentration of 2 mM in β-CD was not used because it did not solubilize. Validation parameters such as the coefficient of determination (R^2^), limit of detection (LD), and limit of quantification (LQ) are included in these results. No interference from methanol or acid water was observed in the spectra.

### 2.3. Solubility Studies

This section reports the solubility of carvedilol in pure DES systems as well as in DES–cyclodextrin combinations. Table 3 summarizes the mean solubility and pH values, together with their corresponding standard deviations, for both the pure systems and those obtained upon cyclodextrin addition using Method 1. In contrast, Table 4 presents the solubility and pH values corresponding to Method 2.

When the solubility values of carvedilol were analyzed using either DES or CDs (α and β), either alone or in combination, both methods showed that the solubility of carvedilol increased as the water content decreased across all the systems studied. This is attributed to the hydrophobic nature of carvedilol. The highest solubility was observed in the choline chloride:xylitol system at a ratio of 2:1:4. The 2:1:10 and 1:2:10 ratios showed the highest solubility in the other systems studied. Furthermore, regardless of the method used, it was found that solubility increased with the amount of cyclodextrin added when cyclodextrin addition was analyzed. This behavior is due to the hydrophobic nature of the cyclodextrin cavity, able to solubilize the hydrophobic carvedilol. The highest solubility was observed with α-CD at a concentration of 2 mM, and an even higher solubility was achieved with β-CD at a concentration of 1 mM.

In addition, various comparisons were carried out to determine which systems were most effective at solubilizing the active ingredient, both on their own and in combination with cyclodextrins.

#### 2.3.1. Solubility of Carvedilol in Pure Solvents Compared with Water

**Figure 2:**
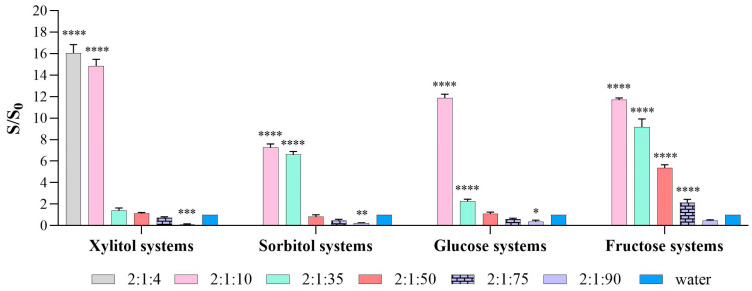
Solubility of carvedilol in DES versus water. The proportions used are 2:1:X, corresponding to 2 parts choline chloride, 1 part sugar, and X parts water. In the case of fructose systems, molar proportions are 1:2:x being x = 10, 35, 50, 75 and 90 of water. Data are expressed as mean ± standard deviation (SD). Statistical significance compared to water was assessed using two-way ANOVA followed by Dunnett’s multiple comparisons test (* *p* < 0.05, ** *p* < 0.01, *** *p* < 0.001. **** *p* < 0.0001).

The solubility of carvedilol was determined in each system and subsequently compared with its solubility in water. The corresponding results are presented in Figure 2. In all DES systems, carvedilol solubility was enhanced relative to water across the evaluated molar ratios (2:1:4, 2:1:10, and 2:1:35). Statistically significant increases in solubility were observed at all ratios, with values markedly higher than those obtained in water (*p* < 0.0001). Among the systems studied, eutectic mixtures based on xylitol and fructose demonstrated the greatest effectiveness in enhancing carvedilol solubility.

In the case of DES containing xylitol, the solubility increases by 16-fold and 14.8-fold for the 2:1:4 and 2:1:10 ratios, respectively. The remaining systems do not significantly increase solubility compared to water; in fact, for the 2:1:75 and 2:1:90 ratios, solubility decreases. In fact, the 2:1:90 ratio differs significantly from water (*p* < 0.001), but the effect is the opposite of what is desired, as it reduces solubility. In fructose-based systems, the highest solubility enhancement was observed at the 1:2:10 ratio, reaching an 11.5-fold increase, followed by the 1:2:35 and 1:2:50 ratios, with 9-fold and 5-fold increases, respectively.

For sorbitol-based systems, the greatest solubility enhancement was obtained with the eutectic mixture at a 2:1:10 ratio, followed by the 2:1:35 ratio, yielding 7.3-fold and 6.8-fold increases, respectively (*p* < 0.0001). In the remaining compositions, solubility decreased relative to that in water. Finally, in glucose-based systems, the 2:1:10 ratio again showed the highest performance, with a 11.9-fold increase in solubility. However, no significant improvement was observed for the 2:1:35 and 2:1:50 ratios. Moreover, at higher water contents, solubility progressively decreased, in some cases falling below that observed in pure water.

#### 2.3.2. Study of the Solubility of Carvedilol in DES Using Methods 1 and 2 with Respect to Water

**Figure 3:**
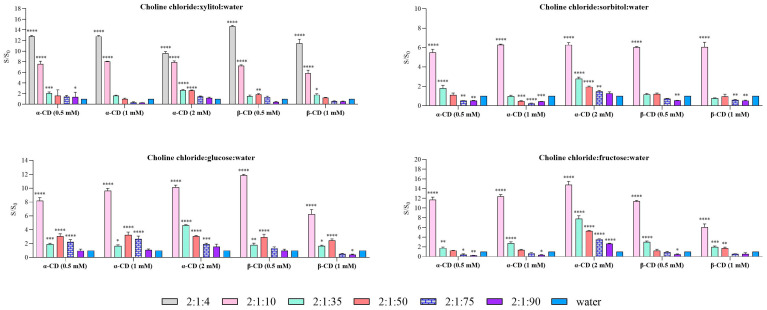
Comparative solubility of carvedilol in cyclodextrin + DES versus water using Method 1. Each graph illustrates a DES for the different ratios that were studied. Data are expressed as mean ± SD. Statistical significance compared to water was assessed using two-way ANOVA followed by Dunnett’s multiple comparisons test (* *p* < 0.05, ** *p* < 0.01, *** *p* < 0.001. **** *p* < 0.0001).

**Figure 4:**
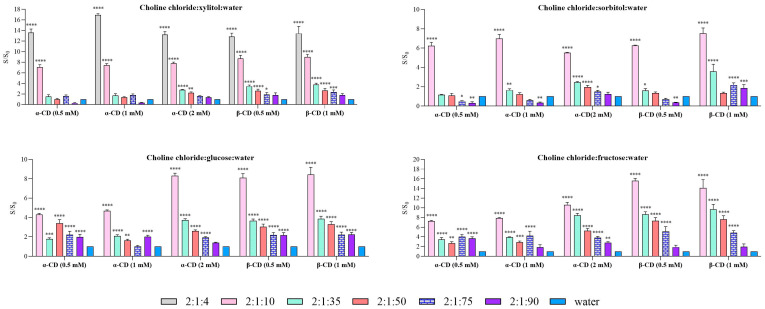
Comparative solubility of carvedilol in cyclodextrin + DES versus water using Method 2. Each graph illustrates a DES for the different ratios that were studied. Data are expressed as mean ± SD. Statistical significance compared to water was assessed using two-way ANOVA followed by Dunnett’s multiple comparisons test (* *p* < 0.05, ** *p* < 0.01, *** *p* < 0.001. **** *p* < 0.0001).

A comparative analysis was performed between the two methods (Method 1 and Method 2) and water in order to identify the most suitable approach and cyclodextrin concentration for enhancing solubilization. The solubility of carvedilol in each DES system and molar ratio is presented in Figure 3 (Method 1) and Figure 4 (Method 2). In both cases, solubility values are expressed relative to water (S/S_0_).

Consistent with the results obtained for pure systems, xylitol-based DES exhibited the highest solubilizing capacity in Method 1 (Figure 3). This trend was maintained upon the addition of cyclodextrins, although a decrease in the solubility ratio was observed as the intrinsic solubility of the added cyclodextrin increased. Notably, the highest enhancement was achieved with β-CD at 0.5 mM, reaching up to a 14.7-fold increase, followed by α-CD at 0.5 and 1 mM, both showing increases of 12.8-fold in the 2:1:4 system. For the 2:1:10 system, solubility enhancement was approximately 7-fold across most cyclodextrin types and concentrations, with the exception of β-CD at 1 mM, which deviated from this trend.

For Method 1 (Figure 3), carvedilol solubility exhibited a clear dependence on DES composition, molar ratio, and the type and concentration of cyclodextrins. Two-way ANOVA revealed significant effects of both DES type and molar ratio, as well as a significant interaction between these factors, indicating that the impact of molar ratio on solubility was dependent on both the cyclodextrin used and the DES composition. In general, DES systems with water molar ratios of 2:1:4 and 2:1:10 showed significantly higher solubility compared to water (*p* < 0.001), and in some cases, systems with a 2:1:35 ratio also exhibited enhanced solubility. However, this pattern was not consistent across all cyclodextrin types and DES compositions, suggesting a non-linear relationship. The incorporation of α- and β-cyclodextrins further altered the solubility profiles, with α-CD demonstrating a more pronounced effect at higher concentrations (2 mM).

In Method 1, and in agreement with the results obtained for pure systems, xylitol-based DES showed the highest solubilizing capacity for carvedilol. This trend was maintained upon the addition of cyclodextrins, although the solubility ratio tended to decrease as the concentration of the added cyclodextrin increased. The highest enhancement was observed with β-CD at 0.5 mM, reaching up to a 14.7-fold increase, followed by α-CD at 0.5 and 1 mM, both yielding 12.8-fold increases in the 2:1:4 system. For the 2:1:10 system, solubility enhancement was approximately 7-fold across most cyclodextrin types and concentrations, with the exception of β-CD at 1 mM.

In sorbitol-based systems, DES containing cyclodextrins (α- and β-CD) exhibited the highest solubility ratios at the 2:1:10 composition, whereas the optimal ratio for the pure DES was 2:1:35. In the 2:1:10 systems with cyclodextrins, solubility ratios were approximately 6-fold in most cases, except for α-CD at 0.5 mM, which showed lower enhancement. The remaining compositions did not show a notable improvement compared to water.

For glucose-based systems, the 2:1:10 ratio again provided the highest solubility enhancement. In the case of α-CD, solubility increased with cyclodextrin concentration, reaching a 10.2-fold increase at 2 mM, compared to 8.2- and 9.7-fold at 0.5 and 1 mM, respectively. For β-CD, the highest solubility ratio (11.9-fold) was comparable to that of the pure system; however, this value decreased markedly at 1 mM.

Finally, in fructose-based systems, the highest solubility enhancement in the presence of cyclodextrins was observed at the 1:2:10 ratio. For α-CD, the solubility ratio increased with concentration, reaching a maximum of 14.8-fold at 2 mM, followed by 11.7- and 12.4-fold at 0.5 and 1 mM, respectively. In contrast, β-CD showed the opposite trend, with decreasing solubility ratios as concentration increased, reaching 11.4-fold at 0.5 mM.

In Method 2 (Figure 4), xylitol-based systems exhibited a trend similar to that observed in Method 1. However, higher solubility was achieved for the 2:1:4 ratio when α-CD was added at a concentration of 1 mM, reaching a 16.9-fold increase. In contrast, for β-CD, the solubility ratios obtained with this method were lower than those observed in Method 1, with values of 12.8-fold and 13.3-fold for β-CD at 0.5 mM and 1 mM, respectively. Across all tested concentrations and for both cyclodextrins, a decrease in solubility was observed for the 2:1:10 system compared to the corresponding pure DES, with solubility ratios ranging between 7- and 9-fold relative to water.

In sorbitol-based systems, Method 2 exhibited a trend comparable to that observed in Method 1, with the 2:1:10 composition providing the highest solubility enhancement. The greatest increases were obtained with α-CD, reaching approximately 7-fold at 1 mM, followed by 6.2-fold at 0.5 mM and 5.5-fold at 2 mM. For β-CD, the highest enhancement was observed at 1 mM, with a solubility increase of 7.5-fold. For the remaining compositions, solubility enhancements were limited, and in some cases, values lower than those observed in water were obtained, likely due to unfavorable intermolecular interactions within the system.

In glucose-based systems, the best performance was again observed at a ratio of 2:1:10; however, the solubility enhancement was markedly lower than that obtained with the corresponding pure DES. The maximum values were 8.3-fold for α-CD at 2 mM and 8.4-fold for β-CD at 1 mM. Interestingly, the solubility ratio for α-CD decreased substantially at lower concentrations (0.5 and 1 mM), approaching approximately half the maximum value.

A similar overall trend was observed for Method 2 (Figure 4). Statistical analysis confirmed significant main effects, as well as a significant interaction between factors, indicating a combined influence of cyclodextrin type and DES molar ratio on carvedilol solubility. However, in contrast to Method 1, the effect of cyclodextrin addition differed, with β-CD showing a greater capacity to enhance carvedilol solubility. This effect was not evident in DES systems with lower water content (2:1:4 and 2:1:10).

For xylitol-based systems, the general trend remained consistent with that observed in Method 1. Nevertheless, higher solubility was achieved at the 2:1:4 ratio when α-CD was added at 1 mM, reaching a 16.9-fold increase. In contrast, β-CD produced lower solubility ratios compared to those obtained in Method 1, with values of 12.8-fold and 13.3-fold at 0.5 mM and 1 mM, respectively. Additionally, across all tested concentrations and for both cyclodextrins, the 2:1:10 system showed a decrease in solubility relative to the corresponding pure DES, with solubility ratios ranging between 7- and 9-fold compared to water.

In Method 2, the sorbitol-based systems exhibited a trend that was broadly similar to that observed in Method 1. The greatest solubility enhancement was obtained for the 2:1:10 composition, with α-CD providing the highest values: a 7-fold increase at 1 mM, compared with 6.2-fold and 5.5-fold at 0.5 and 2 mM, respectively. For β-CD, the maximum increase was observed at 1 mM, reaching 7.5-fold. In the remaining systems, solubility improvements were modest, and in some cases, solubility values were even lower than those obtained in water. This behavior may be attributed to unfavorable intermolecular interactions within the system.

For glucose-based systems, the 2:1:10 ratio again showed the best performance; however, the enhancement was lower than that observed for the corresponding pure DES, reaching a maximum of 8.3-fold for α-CD at 2 mM and 8.4-fold for β-CD at 1 mM. For α-CD, the solubility ratio was reduced by approximately half at 0.5 and 1 mM.

Finally, in the fructose-based systems, the trend relative to the pure DES was altered, as also observed in Method 1. Whereas the pure DES showed the highest solubility at the 1:2:35 composition, the inclusion of cyclodextrin shifted the optimum to the 1:2:10 system. For α-CD, the solubility ratio increased with cyclodextrin concentration, reaching a maximum 10.6-fold enhancement at 2 mM. In contrast, β-CD produced substantially higher increases, with solubility ratios of 15.6 and 14.0 at 0.5 and 1 mM, respectively.

Overall, multiple DES systems improved carvedilol solubility relative to water under both methods, although the magnitude of the effect depended on the DES molar ratio and the presence of cyclodextrins. The differences observed between methods suggest that the experimental procedure has a relevant influence on the final solubility outcomes.

#### 2.3.3. Comparison of the Solubility of Carvedilol in DES + Cyclodextrin with the Solubility of the Active Ingredient in Pure DES

**Figure 5:**
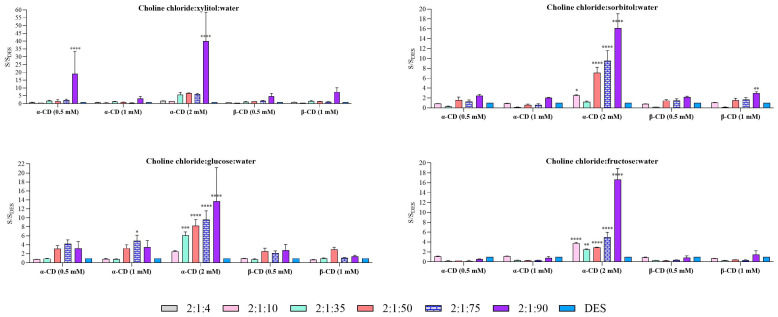
Comparative solubility of carvedilol in DES and cyclodextrin versus DES using Method 1. Each graph illustrates a DES for the different ratios under study. Data are expressed as mean ± SD. Statistical significance compared to water was assessed using two-way ANOVA followed by Dunnett’s multiple comparisons test (* *p* < 0.05, ** *p* < 0.01, *** *p* < 0.001. **** *p* < 0.0001).

**Figure 6:**
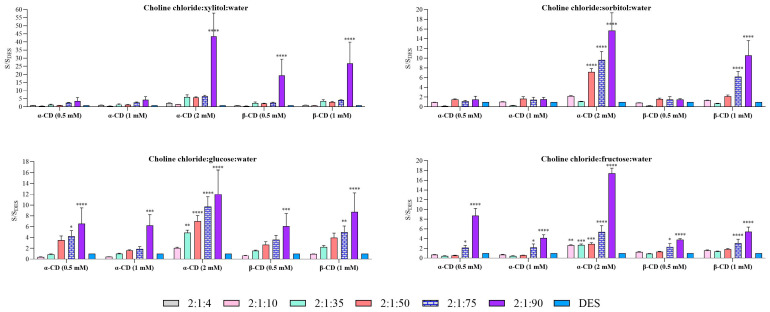
Comparative solubility of carvedilol in DES and cyclodextrin versus DES using Method 2. Each graph illustrates a DES for the different ratios under study. Data are expressed as mean ± SD. Statistical significance compared to water was assessed using two-way ANOVA followed by Dunnett’s multiple comparisons test (* *p* < 0.05, ** *p* < 0.01, *** *p* < 0.001. **** *p* < 0.0001).

To further assess the contribution of cyclodextrins to carvedilol solubility, the results were also analyzed relative to the corresponding DES systems in the absence of cyclodextrins. The effect of cyclodextrin addition, normalized to the matching DES at the same molar ratio, is shown in Figure 5 for Method 1 and Figure 6 for Method 2.

In Method 1, cyclodextrin addition generally produced only limited improvements in carvedilol solubility relative to the corresponding DES systems. Two-way ANOVA revealed significant effects of cyclodextrin concentration and molar ratio, as well as a significant interaction between both factors, indicating that the response depended on system composition. However, significant increases were mainly restricted to α-CD at 2 mM and were predominantly observed in xylitol-, sorbitol-, and glucose-based systems with higher water content (2:1:90), where increases exceeded 10-fold in all cases.

In contrast, Method 2 produced a more pronounced enhancement in carvedilol solubility upon cyclodextrin addition. Statistical analysis confirmed significant main effects of both cyclodextrin concentration and molar ratio, together with a significant interaction between these factors. Notably, a broader range of cyclodextrins yielded significant improvements relative to the corresponding DES systems, particularly under conditions of higher water content.

In xylitol-based systems, Method 1 showed that the highest water content, 2:1:90, provided the greatest solubility enhancement. The largest increase was observed with α-CD at 2 mM, reaching a 31.1-fold improvement relative to the corresponding pure DES. For β-CD, the maximum increase was 6.5-fold in the 2:1:90 system, which, although significant, was markedly lower than that observed for α-CD.

In Method 2, α-CD at 2 mM produced an even greater enhancement, reaching a 38.9-fold increase in solubility, whereas β-CD showed a maximum increase of 22.6-fold at 1 mM. These results highlight the superior solubilizing capacity of α-CD under these conditions compared with β-CD, suggesting differences in complexation efficiency or molecular recognition between the two cyclodextrins.

For sorbitol-based systems, Method 1 showed that α-CD yielded maximum solubility increases of up to 16-fold in the 2:1:90 system. Under the remaining conditions, both α-CD and β-CD produced only modest enhancements of approximately 2-fold, indicating a limited effect outside the optimal composition. In Method 2, the highest increase for α-CD was again observed in the 2:1:90 system, with a 15.5-fold enhancement, followed by the 2:1:75 system, which reached 9.3-fold. For β-CD, the greatest solubility increase also occurred in the 2:1:90 system, with a 10.5-fold enhancement. Overall, these results emphasize that solubility enhancement depends strongly on both the DES composition and the cyclodextrin type used.

In glucose-based systems, Method 1 showed a greater solubility enhancement with α-CD, particularly at 2 mM. At this concentration, the 2:1:90 system exhibited the highest increase in solubility; however, the 2:1:75 and 2:1:50 systems also showed notable enhancements, with increases of 9.4-fold and 8.1-fold, respectively. In contrast, the increases observed with β-CD were less pronounced, indicating lower solubilization efficiency under comparable conditions. Method 2 showed a similar trend to that observed in Method 1. Here, α-CD at 2 mM again produced the greatest solubility enhancement, with an 11-fold increase for the 2:1:90 system and a 9.6-fold increase for the 2:1:75 system. Additional improvements were also observed at other concentrations. A comparable pattern was found for β-CD, although with lower values, reaching a maximum increase of 7.9-fold at 1 mM.

Finally, in fructose-based systems, Method 1 showed a marked increase in the 1:2:90 system, where α-CD produced a 16.5-fold enhancement in solubility relative to the corresponding DES. By contrast, the increases observed with β-CD were less pronounced, suggesting lower effectiveness under these conditions. In Method 2, the highest solubility enhancement was again observed with α-CD at 2 mM, reaching a 17.4-fold increase. β-CD also increased solubility, although to a lesser extent, with a maximum of 5.5-fold at 1 mM. Overall, these findings highlight the superior solubility-enhancing capacity of α-CD compared with β-CD under these conditions.

In both methods, the solubilizing effect of cyclodextrins increased progressively with water content in the DES, underscoring water’s critical role in facilitating cyclodextrin–carvedilol complexation. These findings demonstrate the synergistic influence of DES composition, water content, and cyclodextrin type on carvedilol solubility, while highlighting that the efficiency of cyclodextrin-mediated solubilization is strongly dependent on the experimental methodology employed.

#### 2.3.4. pH Effect

The pH values of carvedilol solutions varied significantly depending on DES composition, molar ratios, and the type and concentration of cyclodextrin (CD) added. Pure DES systems without CD generally exhibited higher pH values, particularly xylitol- and sorbitol-based formulations (reaching pH 8.64 and 8.65, respectively), whereas glucose- and fructose-based systems showed moderately lower values. In all cases, pH decreased progressively with increasing water content in the DES.

In Method 1, the addition of α-CD generally resulted in a slight pH decrease compared to the corresponding pure DES, although this effect was not consistently concentration-dependent across all systems. In contrast, β-CD-containing systems consistently exhibited higher pH values than their α-CD counterparts, in some cases approaching or even exceeding those of the pure DES. The highest pH value recorded (9.11) was observed for the ChCl:Xy:H_2_O (2:1:4) system with 0.5 mM β-CD.

In Method 2, a more pronounced pH decrease was observed upon cyclodextrin addition. Both α-CD and β-CD typically led to acidification across most systems, with pH values ranging from 6.0 to 7.2. This effect was particularly marked in xylitol-based systems, where higher concentrations of α-CD produced notably acidic conditions (e.g., pH 4.83 for ChCl:Xy:H_2_O 2:1:4 with 2 mM α-CD). Overall, pH values obtained with Method 2 were consistently lower than those recorded for Method 1.

#### 2.3.5. Comparison of Carvedilol Solubility Between Method 1 and Method 2

The method of eutectic mixture preparation—either Method 1 (incorporating CD during DES formation) or Method 2 (adding CD with the active ingredient post-DES formation)—substantially influenced carvedilol solubility. These findings suggest that the structural characteristics of the cyclodextrin and the sugar component within the DES are both critical in determining solubilization performance.

The comparison between Method 1 and Method 2, expressed as the solubility ratio (Solubility_method2_/Solubility_method1_), provides direct insight into their relative efficiency. A ratio of 1 indicates equivalent performance between methods, while deviations from unity reflect differences in solubilization capacity. The corresponding results are presented in Figure S1 for each DES system, facilitating visualization of system-specific behavior. A horizontal reference line at a ratio value of 1 was included to clearly identify deviations, where values above or below this threshold indicate superior solubility achieved by Method 2 or Method 1, respectively.

In xylitol-based systems, Method 2 consistently showed higher solubility values compared to Method 1. The greatest enhancement with α-CD was observed at 1 mM in the 2:1:75 system. For other concentrations of α-CD, no significant differences between methods were noted. For β-CD, marked solubility increases were observed in the 2:1:35 system at 0.5 mM (3.8-fold higher than Method 1) and in the 2:1:75 system at 1 mM (4-fold higher), highlighting Method 2’s superior performance for β-CD under these specific conditions.

In sorbitol-based systems, Method 2 showed a modest solubility increase for α-CD at 1 mM. For β-CD, solubility enhancements were more pronounced, with values of 4.5-, 3.7-, and 3.5-fold for the 2:1:35, 2:1:75, and 2:1:90 systems, respectively. These results indicate a moderate but consistent improvement with Method 2 for both cyclodextrins.

In glucose-based systems, Method 2 provided slightly higher solubility for β-CD at higher concentrations (1 mM) in the 2:1:75 and 2:1:90 mixtures, with increases of 4.5- and 5.7-fold, respectively. Solubility improvements in the remaining conditions were not significant, suggesting that Method 2 offers modest benefits primarily for β-CD at elevated concentrations.

In fructose-based systems, Method 2 yielded higher solubility enhancements for both cyclodextrins. For α-CD, the greatest increases were observed at lower concentrations (0.5 mM), reaching 10.5- and 15.7-fold improvements in the 1:2:75 and 1:2:90 systems, respectively. For β-CD, the highest enhancements at 0.5 mM were seen in the 1:2:50 (6.0-fold) and 1:2:75 (5.7-fold) systems, while at 1 mM, the maximum increase (9.1-fold) occurred in the 1:2:75 system. These results demonstrate that Method 2 significantly enhances solubility, particularly at lower cyclodextrin concentrations.

Overall, carvedilol solubility was generally comparable between Method 1 and Method 2 across most conditions. While Method 2 showed enhanced performance under specific DES compositions, molar ratios, and cyclodextrin concentrations, this effect was not consistent throughout. Notably, α-CD at 2 mM produced equivalent solubility values regardless of method, suggesting no clear advantage under these conditions. In contrast, in choline chloride:fructose:water systems at 2:1:75 and 2:1:90 ratios, Method 2 consistently yielded higher solubility ratios (except for α-CD at 2 mM), with β-CD at 1 mM showing particularly marked improvements across all DES systems.

This behavior indicates that α-CD at 2 mM may dominate the solubilization process independently of preparation method, whereas water content critically modulates method-dependent effects when using β-CD at 1 mM.

## 3. Discussion

### 3.1. Influence of pH in Solubility

Carvedilol exhibits complex solubility behavior, which is influenced by the pH gradient of gastrointestinal fluids as it moves from the stomach to the intestine. The drug ionizes and dissolves efficiently in the acidic environment of the stomach. However, when it reaches the more alkaline conditions of the intestine, its solubility decreases, which could lead to precipitation [49].

The chemical structure of carvedilol displays overall weak basic properties with the presence of both acidic and basic functional groups. The carbazole structure contains an NH group that is weakly acidic (pKa 15.0), as the lone pair of electrons participates in aromatization, making the NH group prone to proton dissociation. Additionally, the aliphatic secondary amine in the structure is weakly basic, with a pKa of 7.8 [41,42,50], influenced by the electronegativity of nearby oxygen atoms and steric effects from bulky alkyl groups with aromatic rings. This double ionization behavior allows the formation of a cationic center at acidic pH and an anionic center at basic pH, underpinning its zwitterionic nature and solubility profile [49]. Additionally, based on the Henderson–Hasselbalch equation, carvedilol will exist in its ionized (protonated) form when the pH is lower than its pKa [49]. This behavior can be explained mechanistically by the pH-dependent shift in carvedilol’s ionization equilibrium. At higher pH values, the decrease in protonation results in an increase in the concentration of the less soluble neutral species, thereby limiting its solubility in water.

The solubility of the enantiomers and the racemic mixture was previously evaluated by Zhang et al. [51] in simulated gastrointestinal aqueous media, including deionized water, pH 1.2 HCl solution, and phosphate buffers (pH 2.0–8.0). Across this pH range, solubility values ranged from 7.2 to 882.4 μg/mL for RS-carvedilol, 1.2 to 1523.3 μg/mL for S-carvedilol, and 1.6 to 1695.8 μg/mL for R-carvedilol. Maximum solubility (at pH 2.0) exceeded minimum values (at pH 8.0) by over 1000-fold for all forms. Notably, RS-carvedilol exhibited 1.3–3.2 times lower solubility than its enantiomers at pH 1.2–5.0, but 1.6–7.5 times higher solubility at pH 6.0–8.0. The solubility profiles of S- and R-carvedilol were comparable across the tested pH range [51].

Several studies have characterized carvedilol’s pH-dependent solubility profile. For instance, Yuvaraja et al. reported solubility values of 879 µg/mL at pH 1.2, decreasing markedly to 63.9 µg/mL at pH 6.8 and 30.1 µg/mL at pH 7.4 [52]. Similarly, Hamed et al. demonstrated typical weak base behavior, with low solubility at higher pH (5.8–51.9 μg/mL, pH 6.5–7.8) and substantially higher solubility at acidic pH (545.1–591.4 μg/mL, pH 1.2–5.0) [49].

The pH differences observed among the studied systems can be mainly attributed to the nature of the hydrogen-bond donor in the DES. Xylitol- and sorbitol-based systems consistently generated more basic environments than glucose- and fructose-based DES, which may reflect differences in their hydrogen-bonding networks and, consequently, in their ability to modulate proton availability within the medium. Since carvedilol is a weak base, its protonation is favored under acidic conditions, which can increase its apparent solubility. Therefore, even modest pH shifts may have a marked impact on drug behavior in these systems.

In Method 1, cyclodextrin incorporation produced modest pH modifications, with β-CD consistently generating more basic conditions than α-CD. The pH ranking across systems and cyclodextrins followed the order xylitol > sorbitol > glucose > fructose for α-CD, and xylitol > sorbitol > fructose > glucose for β-CD. This behavior may reflect differences in cavity size and interaction patterns that influence DES organization and proton distribution in solution. However, the effect of CD concentration was not strictly linear, suggesting the involvement of multiple equilibria, including inclusion complex formation and DES structural reorganization.

In contrast, Method 2 produced a more pronounced acidification upon cyclodextrin addition, particularly with α-CD in xylitol-based DES. This indicates that the preparation method critically influences the final physicochemical environment, likely through differences in the extent and nature of interactions between DES components and cyclodextrins. The consistently lower pH values observed with Method 2 suggest greater disruption of the original DES structure or altered proton distribution equilibria.

Salt formation represents the most common strategy for enhancing the apparent intrinsic solubility of acidic and basic drugs. This approach is frequently combined with cyclodextrin complexation to achieve synergistic solubilization effects. Loftsson et al. demonstrated that, at pH values below 5, acetic acid significantly increases carvedilol’s aqueous solubility by forming the more soluble carvedilol acetate salt compared to hydrochloride or phosphate salts. This enhanced solubility improves the complexation efficiency of neutral cyclodextrins such as 2-hydroxypropyl-β-cyclodextrin (HPβCD), thereby reducing the amount of cyclodextrin required for drug solubilization. Additionally, flux studies indicated that carvedilol/HPβCD complexes form water-soluble aggregates in solution [42].

### 3.2. Solubility of Carvedilol in Deep Eutectic Systems Versus Water

The solubility of carvedilol was systematically evaluated in each DES and molar ratio, relative to its solubility in water. In recent years, deep eutectic solvents have gained significant attention as alternative media for enhancing the solubility of hydrophobic drugs, particularly those composed of choline chloride paired with sugar- or polyol-based components. Asgari et al. investigated DES formed by choline chloride and ethylene glycol (1:2), analyzing carvedilol solubility across different temperatures and molar fractions. They observed that solubility increased with both rising temperature and higher DES content. Specifically, in pure DES at 298.15 K, solubility reached 111.3 mg/L, compared to 17.68 mg/L in a 50% DES aqueous solution [53]. Asghar et al. studied the solubility of several APIs—including atenolol, ibuprofen, piroxicam, ketoconazole, carbamazepine, and carvedilol—in aqueous solutions containing DES formed by choline chloride:glycerol (ChCl-G) or choline chloride:urea (ChCl-U) at 10% (ChCl-G10%, ChCl-U10%) and 50% (ChCl-G50%, ChCl-U50%) concentrations. They observed that carvedilol solubility increased proportionally with DES content. Specifically, solubility rose from 32 mg/L in water to 159 mg/L (ChCl-G10%), 873 mg/L (ChCl-G50%), 91 mg/L (ChCl-U10%), and 620 mg/L (ChCl-U50%) [54].

Sayad et al. determined the solubility behavior of carvedilol in aqueous mixtures of a deep eutectic solvent formed by choline chloride and propylene glycol across temperatures ranging from 293.15 to 313.15 K. The solubility data were analyzed using several cosolvency models (van’t Hoff, Yalkowsky, Jouyban-Acree, Jouyban-Acree-van’t Hoff, and modified Wilson models). They observed that carvedilol solubility increased with both rising temperature and DES mass fraction [55]. Our research group previously investigated the solubility and stability of carvedilol in other eutectic systems composed of xylitol, citric acid, glucose, and sorbitol at different molar ratios from those used in the present study. The system exhibiting the highest solubility was citric acid:choline chloride:water (CACh10), which achieved solubility values up to 1506 mg/L [45].

### 3.3. Comparative Analysis of Methods 1 and 2 Versus Water

Carvedilol solubility was systematically evaluated in each DES and molar ratio, both in the presence and absence of cyclodextrins, and compared with its solubility in water. Most DES-CD combinations significantly enhanced carvedilol solubility relative to water, although the magnitude of enhancement varied markedly depending on DES composition, molar ratio, and cyclodextrin type. Notably, systems with lower water content within the DES formulations exhibited the most pronounced solubility improvements, suggesting that an optimal balance between DES structural integrity and controlled hydration is critical for maximizing drug solubilization [56]. Our results demonstrate that DES composition, cyclodextrin type, and preparation method exert a decisive influence on carvedilol solubility relative to water.

Method 1 demonstrated superior solubility enhancement for carvedilol across most eutectic systems, particularly when combined with α-CD. Overall, Method 1 exhibited a stronger synergistic effect between DES and CDs, potentially attributable to its higher pH conditions that favor carvedilol solubilization.

Other researchers have also studied cyclodextrin-based approaches for carvedilol solubility enhancement. Rigaud et al. studied the effects of various CDs on the solubility and chemical stability of carvedilol in aqueous media. They demonstrated that concentrations of 5 mg/mL (12.3 mM) carvedilol could be achieved using 5 equivalents of γ-CD or randomly methylated β-cyclodextrin (RAMEB) in aqueous media at acidic pH (3.5–4.7). Both CDs formed 1:1 inclusion complexes with carvedilol, with RAMEB exhibiting stronger binding affinity compared to γ-CD [57].

Liu et al. analyzed the role of cyclodextrin derivatives in stabilizing carvedilol supersaturation by inhibiting crystallization. Their results showed that β-CD and γ-CD derivatives significantly enhanced carvedilol solubility. Solubilization efficiency was governed by the size compatibility between the drug molecule and the CD cavity. Additionally, the effectiveness of crystallization inhibition was strongly influenced by the type of substituents on the CD derivatives [58].

Shewale et al. studied the influence of pH and hydroxypropyl-β-cyclodextrin (HP-β-CD) concentration on the solubility of carvedilol, which exhibits pH-dependent solubility. Equilibrium solubility was measured across a pH range of 1.2 to 11 and compared to solubility in the presence of 20% HP-β-CD at the same pH values. They found that the solubility of the protonated form of carvedilol was higher than that of the neutral molecule. HP-β-CD increased solubility at all pH levels, although its effectiveness in enhancing solubility may vary depending on the molecule’s charge state. They confirmed that the solubility of carvedilol was enhanced by either inclusion of HP-β-CD or by lowering the pH with various agents. However, when using both methods together, it was crucial to select the pH carefully [43].

Pokharkar et al. studied the use of a ternary complex comprising carvedilol, β-CD, and citric acid to enhance both solubility and dissolution rate. The spray-dried ternary complex achieved a remarkable 110-fold increase in carvedilol solubility compared to the drug alone in water [59].

Zoghbi et al. used two techniques to improve the solubility of carvedilol: complexation with hydroxypropyl-β-cyclodextrin (HPβCD) and solid dispersion (with Poloxamer 188 (PLX) and Polyvinylpyrrolidone K-30 (PVP)). The inclusion complex (KD) and the dispersions were developed using different methods. Solubility and dissolution were analyzed in three media. The solid dispersions exhibited solubility increases of up to 70-fold in double-distilled water, 25-fold in simulated gastric fluid, and 22-fold in phosphate-buffered saline compared to pure carvedilol in these media [60].

The results obtained in this study are supported by the previous literature, which consistently reports enhanced carvedilol solubility relative to aqueous conditions, primarily attributed to medium acidification that favors salt formation and drug protonation [57]. The solubilization process involves the formation of 1:2 molar ratio inclusion complexes with β-CD, confirmed by FTIR, H-NMR, XRD, and phase solubility studies. Additionally, the structure of the inclusion complex was characterized by fluorescence spectroscopy, where enhancement in fluorescence intensity suggested that carvedilol resides within the hydrophobic cavity of cyclodextrins and remains stable [61,62].

### 3.4. Comparative Analysis of Solubility Values in Methods 1 and 2 Versus DES

To further assess the specific contribution of cyclodextrins to carvedilol solubilization, we compared the effect of DES systems with and without CDs for Methods 1 and 2. This approach allows us to evaluate the performance of cyclodextrins independently within each experimental methodology.

α-CD primarily forms inclusion complexes with aliphatic hydrocarbons, whereas β-CD is more effective for smaller aromatic molecules and is widely utilized due to its ability to enhance aqueous solubility, ready availability, and low cost. In contrast, γ-CD is better suited for larger molecules. The characteristic hydrophilic outer surface and lipophilic inner cavity of CDs enable the encapsulation of lipophilic drugs, forming stable inclusion complexes [63].

The heights of the α and β CD cavities are uniform at 7.9 Å while their diameters range from 4.7 to 5.3 Å and 6.0 to 6.5 Å, with volumes of 174 and 262 Å^3^, respectively [64]. These cone-shaped molecules show a hydrophilic outer surface and hydrophobic inner cavity that promote the formation of inclusion complexes in aqueous environments with carvedilol, characterized by low hydrophilicity and suitable volume to facilitate intermolecular interactions. It is worth mentioning that xylitol shows the smaller polar surface area (101 Å2) followed by glucose, fructose and sorbitol, with 110, 118 and 121 Å^2^, respectively (ACDLabs Chemspider). Polar surface area of carvedilol is estimated to be 76 Å^2^ (ACDLabs Chemspider) while molecular volume calculated from molar refraction (hard-core of the molecule) is 198.57 Å^3^ (ACDLabs Chemspider).

It should therefore be noted that DES components can interact strongly with cyclodextrins, which could result in competition for the formation of inclusion complexes with the drug. This was evident from the lack of improvement in solubilization observed for almost all of the cyclodextrins tested using Method 1. In this context, it has been reported that the high affinity of DES constituents for cyclodextrins can reduce the effective formation of drug–cyclodextrin complexes, leading to lower-than-expected solubilization enhancements [65]. In fact, Kfoury et al. point out that the high solubility of CDs in DES may not necessarily translate into improved drug solubility via complex formation [66].

In contrast, Method 2 (Figure 6) exhibited a more pronounced effect from cyclodextrin addition, yielding higher solubility values in several DES systems compared to their CD-free counterparts. This enhancement was particularly evident in DES formulations with higher water content, suggesting that increased hydration facilitates carvedilol–cyclodextrin complexation.

Water plays a critical role in both forming and stabilizing inclusion complexes by modulating the system’s microenvironment and transport properties. In higher-water-content media, reduced effective viscosity enhances molecular mobility and diffusion, increasing solute–solvent encounter probability. This effect is particularly relevant for cyclodextrin systems, where water participates in essential hydration/dehydration equilibria required for complex formation and stability. Collectively, these factors position water as an active component that not only facilitates diffusion-mediated complexation but also stabilizes the resulting inclusion complexes [67,68].

Overall, these findings demonstrate that cyclodextrin addition’s effect on carvedilol solubility is highly dependent on both DES composition and experimental method. While cyclodextrins do not universally enhance solubility relative to DES alone, their effectiveness is maximized under conditions favoring reduced viscosity and minimized competitive interactions between DES components and cyclodextrins.

### 3.5. Relative Carvedilol Solubility Analysis of Method 1 and Method 2

The solubility ratio between Method 1 and Method 2 provides direct insight into how the experimental approach influences solubilization efficiency of DES systems containing cyclodextrins.

The solubility ratio’s dependence on DES water content further supports the fact that molecular mobility and solvent structure significantly modulate experimental method effectiveness. This aligns with previous studies documenting water’s profound influence on DES viscosity and hydrogen-bond network structure, which directly impacts drug solubilization capacity [69,70]. In more hydrated systems, enhanced diffusion and reduced viscosity likely facilitate carvedilol–cyclodextrin and carvedilol–DES interactions. This explains Method 2’s superior performance with β-CD—despite its relatively low water solubility among native cyclodextrins —across all DES systems at higher water contents [71]. In contrast, the comparable performance of both methods with α-CD at high concentrations suggests method-independent solubilization dominated by α-CD’s intrinsic properties. This interpretation is consistent with reports of cyclodextrin inclusion complexes in DES exhibiting self-association into dimers and higher-order aggregates, substantially enhancing guest solubility [65].

## 4. Materials and Methods

### 4.1. Chemicals

Information on the pure chemicals used in this study is summarized in Table 5. All chemicals were dried under vacuum 24 h before use.

### 4.2. Preparation of Natural Deep Eutectic Solvents

The DES are synthesized using choline chloride, different sugars (xylitol, sorbitol glucose or fructose) and ultra-pure water at different proportions (4, 10, 35, 50, 75 and 90). The components are weighed on a balance, and the mixture is heated to 60–80 °C with constant stirring until a clear liquid is formed. These systems have been previously analyzed and characterized by our research group [24].

### 4.3. Solubility Study

**Figure 7:**
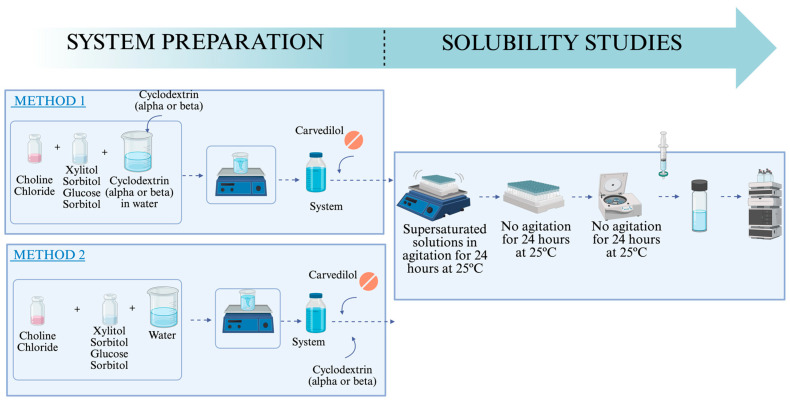
Solubility study using Methods 1 and 2 preparation system. Created with https://BioRender.com.

The solubility was determined using the modified shake-flask method [72]. The solubility of carvedilol was measured in the 21 pure systems, and in addition, two different cyclodextrins were used, α and β, with different concentrations, including α-CD 0.5 mM, 1 mM and 2 mM; and β-CD 0.5 mM and 1 mM. Two different methods were used to incorporate the CDs in the studied systems (Figure 7).

In Method 1, cyclodextrin was added to the water of the DES, previous to the formation of the eutectic mixture. In Method 2, cyclodextrin was directly added to the DES.

The solubility study was performed three times with DES, DES with cyclodextrin, and ultra-pure water, preparing supersaturated carvedilol solutions. Supersaturated solutions were prepared in accordance with standard protocols, and their supersaturation was verified by visual inspection. The samples were stirred for 24 h using a Comecta-Ivymen orbital agitator, followed by an additional 24 h of rest without agitation at a controlled temperature of 25 °C. Afterward, the samples were centrifuged at 4500 rpm for 5 min using a Bunsen Hemoplus centrifuge (Madrid, Spain). The supernatants were then filtered through a 0.22 μm polyethersulfone syringe filter, and carvedilol concentration was determined by High-Performance Liquid Chromatography with Diode Array Detection (HPLC-DAD) on an Agilent 1220 DAD instrument (Waldbronn, Germany). The analysis was conducted using an ODS column (5 mm × 250 mm × 4 mm), with a 20 µL injection volume, a flow rate of 1 mL/min, and a temperature of 25 °C from Analysis Vinílicos^®^ (Cuidad Real, Spain). The isocratic mobile phase consisted of acidified water at pH 4 and methanol in a 30:70 ratio, with the detection wavelength for carvedilol set at 242 nm.

### 4.4. Statistical Analysis

All experiments were performed in triplicate, and results are expressed as the mean ± standard deviation. Statistical analyses were carried out using GraphPad Prism version 11.0.0 (GraphPad Software, San Diego, CA, USA). The effect of DES and molar ratio on the solubility of carvedilol was evaluated in comparison with water, as well as in the presence of cyclodextrins (α- and β-cyclodextrin) at different concentrations and in comparison with DES. Carvedilol solubility data were analyzed using two-way analysis of variance (two-way ANOVA), considering DES type and molar ratio as fixed factors and including their interaction term to assess potential interdependencies between variables. When appropriate, solubility values were compared against water or DES as the reference. When significant effects were observed, Dunnett’s multiple comparisons test was applied to compare each condition against water within the same molar ratio.

To further compare the performance of the two solubility determination methods, the results were expressed as a solubility ratio (Method 2/Method 1). The statistical significance of this ratio relative to unity was assessed using a one-sample *t*-test. A value of 1 indicates no difference between the two methods. A *p*-value < 0.05 was considered statistically significant.

## 5. Conclusions

In this study, the solubility of the active ingredient carvedilol was investigated in 21 deep eutectic systems formed by choline chloride and sugars (xylitol, sorbitol, glucose, and fructose), both individually and in combination with α- and β-CD at different concentrations and two different methods (1 and 2) for incorporating CD in the mixtures.

While the solubility of carvedilol is affected by pH shifts in both methods analyzed, certain systems, particularly those involving xylitol and sorbitol, demonstrate potential for maintaining more basic pH values. In Method 1, the xylitol-based system demonstrates the most effective solubilization, achieving up to a 16-fold increase in solubility, with β-CD further enhancing solubility, particularly at lower concentrations. Sorbitol- and glucose-based systems show more modest improvements, with sorbitol-based systems performing best at a 2:1:10 ratio and glucose-based systems showing enhanced solubility primarily in the 2:1:10 ratio. For fructose-based systems, solubility is most improved in the 2:1:35 ratio without cyclodextrins and increases when β-CD is added. Method 2 reveals similar trends, with some variations, such as higher solubility observed for xylitol and sorbitol systems at 1 mM α-CD and a decrease in solubility for β-CD as its concentration increases.

The addition of α and β cyclodextrins to the eutectic systems significantly enhances the solubility of carvedilol, with the most pronounced improvements observed in systems with the highest water content. Across all methods studied, α-CD consistently outperforms β-CD in terms of solubility enhancement. Notably, xylitol, sorbitol, and glucose-based systems at the 2:1:90 ratio, and fructose-based systems at the 1:2:90 ratio, show the greatest solubility increases, with α-CD achieving up to a 38.9-fold increase in solubility in Method 2. β-CD also improves solubility, but to a lesser extent, with the highest increase observed in xylitol-based systems (22.6-fold in Method 2). These results demonstrate the critical role of cyclodextrin type, concentration, and system composition in optimizing solubility, highlighting the superior solubility-enhancing capabilities of α-CD. The findings provide valuable insights for the formulation of carvedilol in pharmaceutical systems, suggesting that specific cyclodextrin and DES combinations can be tailored to achieve optimal drug solubility.

From a theoretical standpoint, the solubility enhancement can be rationalized by the interplay between pH-dependent ionization of carvedilol, which governs its intrinsic aqueous solubility, and the supramolecular interactions provided by cyclodextrins. In parallel, the structured hydrogen bonding network and polarity modulation of the deep eutectic systems likely contribute to improved drug stabilization in solution, thereby reducing precipitation and favoring the dissolved state.

This study presents some limitations that should be acknowledged. In particular, the lack of molecular characterization limits a deeper understanding of the specific interactions governing the observed solubility enhancements, and the results are based on equilibrium solubility measurements under controlled conditions, which may not fully represent in vivo environments. Future work should focus on incorporating complementary techniques, such as spectroscopic or computational studies, to elucidate specific interactions, as well as evaluating the performance of these systems under biorelevant conditions to further assess their pharmaceutical applicability.

## Figures and Tables

**Table 1:** Composition, molar ratio, molecular weight, density and appearance at different times of the studied DES. Values of density DES are taken from our previous paper [24].

DES	Ratio	Molecular Mass (g/mol)	Density 25 °C (g/mL)	Physical Appearance (Qualitative)
ChCl:Xy:H_2_O	2:1:4	71.91	1.186	Transparent, viscous
	2:1:10	47.03	1.153	Transparent, viscous
	2:1:35	27.93	1.082	Transparent, viscous
	2:1:50	25.12	1.064	Transparent, non-viscous
	2:1:75	22.84	1.044	Transparent, non-viscous
	2:1:90	22.06	1.043	Transparent, non-viscous
ChCl:Sor:H_2_O	2:1:10	49.34	1.234	Transparent, viscous
	2:1:35	28.72	1.196	Transparent, viscous
	2:1:50	25.69	1.122	Transparent, non-viscous
	2:1:75	23.22	1.101	Transparent, non-viscous
	2:1:90	22.38	1.085	Transparent, non-viscous
ChCl:Glu:H_2_O	2:1:10	49.18	1.220	Transparent, viscous
	2:1:35	28.67	1.175	Transparent, viscous
	2:1:50	25.65	1.099	Transparent, non-viscous
	2:1:75	23.20	1.079	Transparent, non-viscous
	2:1:90	22.36	1.056	Transparent, non-viscous
ChCl:Fru:H_2_O	1:2:10	52.30	1.279	Transparent, viscous
	1:2:35	29.73	1.157	Transparent, viscous
	1:2:50	26.41	1.123	Transparent, non-viscous
	1:2:75	23.18	1.120	Transparent, non-viscous
	1:2:90	22.79	1.077	Transparent, non-viscous

ChCl: choline chloride; Xy: xylitol; Sor: sorbitol; Glu: glucose and Fru: fructose.

**Table 2:** Calibration equation carvedilol in acid water/methanol. Wavelength of maximum absorbance (Abs), λ Abs_max_, and validation parameters: coefficient of determination, R^2^, limit of detection, LD, and limit of quantification, LQ. ^a^ $LD=\frac{x+3S}{m}$; ^b^ $LQ=\frac{x+10S}{m}$ being m the slope, and x and S the average and the deviation of the blank.

API	Slope m (mg/L)	λ (Abs_max_) (nm)	R^2^	LD ^a^	LQ ^b^
Carvedilol	133.75	242	0.9998	1.31 × 10^−7^	3.55 × 10^−7^
Carvedilol and α-CD 0.5 mM	131.06	242	0.9997	3.03 × 10^−6^	4.64 × 10^−6^
Carvedilol and α-CD 1 mM	122.57	242	0.9998	4.46 × 10^−6^	8.73 × 10^−6^
Carvedilol and α-CD 2 mM	55.556	242	0.9996	3.64 × 10^−6^	7.34 × 10^−6^
Carvedilol and β-CD 0.5 mM	139.08	242	0.9992	1.08 × 10^−8^	8.48 × 10^−6^
Carvedilol and β-CD 1 mM	98.141	242	0.9992	7.67 × 10^−6^	1.09 × 10^−5^

m: slope of the calibration equation.

**Table 3:** Solubility, s (mg/L), and pH results with their corresponding standard deviation for carvedilol in studied DES and water using Method 1.

Deep Eutectic Systems	Molar Ratio	Only DES		α-CD Concentration						β-CD Concentration			
				0.5 mM		1 mM		2 mM		0.5 mM		1 mM	
		*s*	pH	*s*	pH	*s*	pH	*s*	pH	*s*	pH	*s*	pH
ChCl:Xy:H_2_O	2:1:4	190.7 ± 1.33	8.64 ± 0.13	169.9 ± 5.09	8.59 ± 0.10	163.9 ± 1.01	8.56 ± 0.04	336.7 ± 3.68	6.76 ± 0.01	166.9 ± 3.75	9.11 ± 0.04	178.8 ± 7.47	8.72 ± 0.07
	2:1:10	176.3 ± 1.69	8.26 ± 0.11	100.4 ± 3.93	8.31 ± 0.01	103.4 ± 2.28	8.51 ± 0.01	278.0 ± 2.83	7.17 ± 0.05	82.57 ± 1.37	8.68 ± 0.04	91.24 ± 0.61	8.22 ± 0.03
	2:1:35	16.61 ± 3.17	7.84 ± 0.06	27.79 ± 2.17	7.78 ± 0.04	20.80 ± 1.46	8.11 ± 0.03	93.45 ± 5.29	7.51 ± 0.02	17.20 ± 2.09	8.20 ± 0.05	27.50 ± 0.69	7.83 ± 0.04
	2:1:50	13.65 ± 0.76	7.54 ± 0.12	21.16 ± 13.9	7.54 ± 0.02	12.61 ± 1.94	7.80 ± 0.06	91.13 ± 2.91	7.55 ± 0.03	20.96 ± 1.34	7.98 ± 0.07	19.13 ± 1.09	7.62 ± 0.04
	2:1:75	8.730 ± 1.25	7.53 ± 0.15	18.89 ± 3.34	7.35 ± 0.02	4.390 ± 1.48	7.65 ± 0.04	52.02 ± 3.34	7.79 ± 0.02	15.37 ± 1.62	7.72 ± 0.08	9.290 ± 0.20	7.34 ± 0.02
	2:1:90	1.250 ± 0.59	7.59 ± 0.08	18.29 ± 11.0	7.18 ± 0.02	3.690 ± 0.11	7.54 ± 0.06	42.57 ± 5.61	7.81 ± 0.07	5.290 ± 1.01	7.64 ± 0.02	8.160 ± 0.45	7.17 ± 0.06
ChCl:Sor:H_2_O	2:1:10	86.55 ± 1.84	8.65 ± 0.45	73.10 ± 1.91	8.53 ± 0.04	80.63 ± 0.58	8.20 ± 0.03	220.4 ± 3.91	7.10 ± 0.03	68.57 ± 1.33	8.49 ± 0.01	94.30 ± 2.37	8.11 ± 0.02
	2:1:35	78.67 ± 4.50	7.83 ± 0.01	23.94 ± 3.34	7.93 ± 0.05	12.65 ± 1.15	7.88 ± 0.02	97.94 ± 6.00	7.36 ± 0.02	13.18 ± 1.35	7.98 ± 0.03	12.34 ± 1.42	7.45 ± 0.03
	2:1:50	9.780 ± 1.85	7.45 ± 0.07	15.05 ± 2.95	7.78 ± 0.07	6.020 ± 0.84	7.64 ± 0.03	68.59 ± 3.30	7.83 ± 0.03	13.97 ± 0.69	7.71 ± 0.04	14.81 ± 3.02	7.52 ± 0.05
	2:1:75	5.690 ± 1.50	7.15 ± 0.02	6.900 ± 0.06	7.41 ± 0.06	3.220 ± 0.35	7.43 ± 0.02	52.10 ± 2.68	7.79 ± 0.05	8.120 ± 0.29	7.55 ± 0.02	9.230 ± 0.19	7.49 ± 0.02
	2:1:90	2.800 ± 0.18	7.16 ± 0.02	6.870 ± 0.20	7.18 ± 0.03	5.740 ± 0.19	7.29 ± 0.04	44.91 ± 5.30	7.91 ± 0.09	6.180 ± 0.06	7.58 ± 0.01	8.340 ± 0.21	7.45 ± 0.05
ChCl:Glu:H_2_O	2:1:10	141.37 ± 7.08	7.96 ± 0.05	109.0 ± 2.39	7.76 ± 0.03	123.8 ± 5.99	8.01 ± 0.02	356.3 ± 4.99	6.74 ± 0.03	135.0 ± 1.63	6.64 ± 0.02	97.31 ± 1.09	9.01 ± 0.07
	2:1:35	27.11 ± 3.14	7.40 ± 0.16	25.31 ± 1.68	7.70 ± 0.02	21.27 ± 1.68	7.79 ± 0.11	163.4 ± 4.07	7.27 ± 0.01	20.64 ± 2.45	6.93 ± 0.08	26.03 ± 1.38	8.84 ± 0.05
	2:1:50	13.21 ± 2.02	7.41 ± 0.06	40.66 ± 3.46	6.20 ± 0.02	41.56 ± 4.79	7.59 ± 0.03	107.2 ± 3.41	7.38 ± 0.03	33.22 ± 4.18	5.98 ± 0.07	38.08 ± 1.32	8.41 ± 0.07
	2:1:75	7.110 ± 0.96	7.15 ± 0.03	29.62 ± 3.53	6.65 ± 0.01	34.20 ± 4.83	7.52 ± 0.01	67.08 ± 5.91	7.69 ± 0.04	15.02 ± 1.88	6.61 ± 0.04	7.690 ± 1.51	8.08 ± 0.02
	2:1:90	4.460 ± 1.59	7.12 ± 0.06	12.70 ± 2.90	6.72 ± 0.03	14.02 ± 1.33	7.46 ± 0.04	54.06 ± 12.4	7.82 ± 0.02	11.01 ± 2.38	6.90 ± 0.04	6.110 ± 1.05	7.99 ± 0.05
ChCl:Fru:H_2_O	1:2:10	139.4 ± 6.88	7.12 ± 0.19	156.0 ± 2.20	6.72 ± 0.03	159.3 ± 5.97	6.90 ± 0.02	519.9 ± 13.5	6.36 ± 0.04	129.5 ± 3.36	7.12 ± 0.03	94.59 ± 4.56	7.02 ± 0.03
	1:2:35	198.9 ± 4.63	6.47 ± 0.06	23.38 ± 2.52	7.18 ± 0.03	35.53 ± 2.73	6.42 ± 0.10	274.2 ± 18.4	6.85 ± 0.05	34.19 ± 1.77	6.82 ± 0.05	31.07 ± 0.86	6.76 ± 0.03
	1:2:50	63.63 ± 1.22	6.46 ± 0.10	16.40 ± 0.20	6.81 ± 0.01	17.88 ± 1.05	6.29 ± 0.02	186.2 ± 6.18	7.12 ± 0.02	13.90 ± 3.71	6.85 ± 0.02	26.71 ± 0.98	6.96 ± 0.07
	1:2:75	28.83 ± 2.77	6.76 ± 0.18	5.050 ± 2.24	7.10 ± 0.03	8.740 ± 1.48	6.37 ± 0.01	124.6 ± 5.2	7.41 ± 0.05	10.08 ± 2.08	6.90 ± 0.02	8.300 ± 0.51	6.86 ± 0.02
	1:2:90	5.680 ± 0.76	6.46 ± 0.02	3.110 ± 0.17	7.26 ± 0.10	4.460 ± 0.82	6.41 ± 0.01	93.74 ± 4.50	7.84 ± 0.08	4.870 ± 1.33	6.94 ± 0.01	8.480 ± 5.38	6.94 ± 0.03
Water		11.90 ± 0.50	7.41 ± 0.07	13.29 ± 0.43	6.89 ± 0.04	12.83 ± 0.22	7.60 ± 0.04	35.11 ± 0.91	7.04 ± 0.05	11.37 ± 0.25	7.69 ± 0.16	15.67 ± 1.62	7.20 ± 0.09

ChCl: choline chloride; Xy: xylitol; Sor: sorbitol; Glu: glucose, and Fru: fructose.

**Table 4:** Solubility, s (mg/L), and pH results with their corresponding standard deviation for carvedilol in studied DES and water using Method 2.

Deep Eutectic Systems	Molar Ratio	Only DES		α-CD Concentration						β-CD Concentration			
				0.5 mM		1 mM		2 mM		0.5 mM		1 mM	
		*s*	pH	*s*	pH	*s*	pH	*s*	pH	*s*	pH	*s*	pH
ChCl:Xy:H_2_O	2:1:4	190.7 ± 1.33	8.64 ± 0.13	180.9 ± 4.91	6.95 ± 0.06	217.2 ± 4.82	6.84 ± 0.02	463.2 ± 10.49	4.83 ± 0.07	145.8 ± 8.05	5.56 ± 0.01	209.0 ± 3.73	6.12 ± 0.03
	2:1:10	176.3 ± 1.69	8.26 ± 0.11	93.59 ± 3.81	6.34 ± 0.06	95.40 ± 4.26	6.57 ± 0.04	274.2 ± 0.882	6.52 ± 0.12	98.92 ± 5.45	6.02 ± 0.02	140.2 ± 6.32	6.37 ± 0.02
	2:1:35	16.61 ± 3.17	7.84 ± 0.06	20.26 ± 4.59	6.41 ± 0.03	22.35 ± 4.15	6,70 ± 0.04	97.82 ± 4.518	6.84 ± 0.02	39.47 ± 1.80	6.20 ± 0.02	58.66 ± 2.05	6.16 ± 0.04
	2:1:50	13.65 ± 0.76	7.54 ± 0.12	13.31 ± 1.42	6.28 ± 0.02	17.55 ± 1.32	6.58 ± 0.01	77.88 ± 5.133	6.97 ± 0.04	29.36 ± 3.23	6.43 ± 0.01	41.32 ± 5.68	6.45 ± 0.02
	2:1:75	8.730 ± 1.25	7.53 ± 0.15	21.24 ± 2.28	6.46 ± 0.04	22.93 ± 2.64	6.37 ± 0.06	56.29 ± 2.160	7.09 ± 0.02	21.30 ± 4.23	6.49 ± 0.05	37.15 ± 4.96	6.80 ± 0.08
	2:1:90	1.250 ± 0.59	7.59 ± 0.08	3.733 ± 1.12	6.00 ± 0.07	4.748 ± 0.48	6.97 ± 0.07	48.60 ± 3.119	7.14 ± 0.02	20.27 ± 4.33	6.65 ± 0.03	28.25 ± 4.83	6.93 ± 0.01
ChCl:Sor:H_2_O	2:1:10	86.55 ± 1.84	8.65 ± 0.45	82.88 ± 2.18	7.18± 0.04	89.54 ± 4.78	7.22 ± 0.03	194.2 ± 4.314	6.43 ± 0.09	71.15 ± 1.53	5.77 ± 0.01	117.4 ± 4.58	5.48 ± 0.08
	2:1:35	78.67 ± 4.50	7.83 ± 0.01	15.38 ± 0.62	6.04 ± 0.05	21.08 ± 1.55	6.06 ± 0.04	86.11 ± 1.739	6.08 ± 0.03	18.28 ± 2.43	5.90 ± 0.03	55.40 ± 6.61	6.20 ± 0.01
	2:1:50	9.780 ± 1.85	7.45 ± 0.07	14.53 ± 2.42	6.08 ± 0.02	15.77 ± 2.20	6.16 ± 0.01	69.27 ± 7.26	6.36 ± 0.03	15.43 ± 1.14	5.81 ± 0.01	21.07 ± 1.73	6.08 ± 0.02
	2:1:75	5.690 ± 1.50	7.15 ± 0.02	6.452 ± 0.96	6.15 ± 0.10	7.856 ± 1.29	6.16 ± 0.04	53.14 ± 5.11	6.50 ± 0.02	7.92 ± 1.43	5.88 ± 0.08	34.30 ± 4.61	6.30 ± 0.09
	2:1:90	2.800 ± 0.18	7.16 ± 0.02	4.206 ± 1.66	5.88 ± 0.22	4.286 ± 1.05	6.20 ± 0.04	43.46 ± 7.80	6.51 ± 0.04	4.178 ± 0.32	5.92 ± 0.05	29.28 ± 6.96	6.42 ± 0.01
ChCl:Glu:H_2_O	2:1:10	141.4 ± 7.08	7.96 ± 0.05	57.22 ± 3.01	5.60 ± 0.03	60.27 ± 0.35	5.75 ± 0.04	292.2 ± 6.70	6.34 ± 0.02	92.02 ± 3.64	5.49 ± 0.07	131.7 ± 4.48	5.89 ± 0.04
	2:1:35	27.11 ± 3.14	7.40 ± 0.16	23.72 ± 0.77	5.67 ± 0.05	26.80 ± 0.22	5.96± 0.03	131.5 ± 1.775	6.34 ± 0.02	41.63 ± 1.17	6.44 ± 0.04	60.58 ± 2.11	6.34 ± 0.04
	2:1:50	13.21 ± 2.02	7.41 ± 0.06	45.23 ± 3.49	5.77 ± 0.10	21.06 ± 1.82	6.32 ± 0.02	91.32 ± 2.64	6.56 ± 0.03	34.56 ± 2.42	6.11 ± 0.06	51.47 ± 3.41	6.29 ± 0.07
	2:1:75	7.110 ± 0.96	7.15 ± 0.03	29.46 ± 3.87	6.02 ± 0.05	13.07 ± 1.10	6.47 ± 0.04	67.91 ± 3.346	6.52 ± 0.03	25.05 ± 2.13	6.20 ± 0.05	34.56 ± 3.46	5.83 ± 0.12
	2:1:90	4.460 ± 1.59	7.12 ± 0.06	26.33 ± 3.35	6.28 ± 0.09	25.71 ± 2.02	6.41 ± 0.05	48.84 ± 2.556	6.32 ± 0.09	24.61 ± 2.48	6.44 ± 0.05	35.12 ± 4.25	6.39 ± 0.02
ChCl:Fru:H_2_O	1:2:10	139.4 ± 6.88	7.12 ± 0.19	96.89 ± 2.01	5.67 ± 0.04	101.1 ± 1.66	5.84 ± 0.08	373.6 ± 10.18	6.22 ± 0.03	177.6 ± 1.65	5.88 ± 0.05	219.8 ± 8.92	5.36 ± 0.04
	1:2:35	198.9 ± 4.63	6.47 ± 0.06	46.35 ± 6.04	6.20 ± 0.02	50.15 ± 1.90	6.21 ± 0.03	296.7 ± 7.192	6.54 ± 0.08	98.33 ± 5.21	6.50 ± 0.05	150.9 ± 1.51	5.77 ± 0.04
	1:2:50	63.63 ± 1.22	6.46 ± 0.10	35.79 ± 2.95	6.00 ± 0.15	36.89 ± 3.09	6.22 ± 0.04	185.1 ± 18.63	6.58 ± 0.01	83.21 ± 5.66	6.43 ± 0.03	118.6 ± 7.98	6.50 ± 0.04
	1:2:75	28.83 ± 2.77	6.76 ± 0.18	53.22 ± 4.94	6.24 ± 0.04	54.32 ± 1.11	6.04 ± 0.10	134.4 ± 11.51	6.74 ± 0.01	57.69 ± 10.3	6.36 ± 0.06	75.70 ± 9.48	6.39 ± 0.02
	1:2:90	5.680 ± 0.76	6.46 ± 0.02	48.88 ± 3.77	6.31 ± 0.05	23.77 ± 6.18	6.36 ± 0.03	98.58 ± 8.442	6.59 ± 0.02	21.50 ± 4.15	6.57 ± 0.23	31.26 ± 8.77	6.82 ± 0.04
Water		11.90 ± 0.50	7.41 ± 0.07	13.29 ± 0.43	6.89 ± 0.04	12.83 ± 0.22	7.60 ± 0.04	35.11 ± 0.91	7.04 ± 0.05	11.37 ± 0.25	7.69 ± 0.16	15.67 ± 1.62	7.20 ± 0.09

ChCl: choline chloride; Xy: xylitol; Sor: sorbitol; Glu: glucose, and Fru: fructose.

**Table 5:** Information on the pure chemicals used in this study.

Chemical	CAS Number	Molar Mass (g/mol)	Supplier	Formula
Xylitol	87-99-0	152.15	Fagron Iberica (Barcelona, Spain)	C_5_H_12_O_5_
Fructose	57-48-7	180.16	Panreac (Barcelona, Spain)	C_6_H_12_O_6_
Glucose anhydrous	50-99-7	180.16	Acofarma (Barcelona, Spain)	C_6_H_12_O_6_
Sorbitol	50-70-4	182.17	Sigma-Aldrich (Darmstadt, Germany)	C_6_H_14_O_6_
Choline Chloride	67-48-1	139.63	Panreac (Barcelona, Spain)	C_5_H_14_ClNO
Carvedilol	72956-09-3	406.5	Acofarma (Barcelona, Spain)	C_24_H_26_N_2_O_4_
α-CD	10016-20-3	972.86	Glentham Life Sciences (Corsham, UK)	C_36_H_60_O_30_
β-CD	7585-39-9	1134.98	Thermoscientific (Waltham, MA, USA)	C_42_H_70_O_35_

## Data Availability

No new data were created or analyzed in this study. Data sharing is not applicable to this article.

## Supplementary Materials

The following supporting information can be downloaded at: https://www.mdpi.com/article/10.3390/molecules31091490/s1, Figure S1: Solubility ratio of carvedilol obtained using Method 2 relative to Method 1 in DES and molar ratios through each cyclodextrins.
