# Supplementary material for: Sugar-Based Eutectic Systems Combined with Cyclodextrins for Enhanced Solubility of Carvedilol
Source: Molecules. 2026 Apr 29;31(9):1490. doi: 10.3390/molecules31091490 (PMC13164601; doi:10.3390/molecules31091490)
Supplement: Supplementary file 1 [file molecules-31-01490-s001.zip › molecules-4238918-supplementary.pdf]

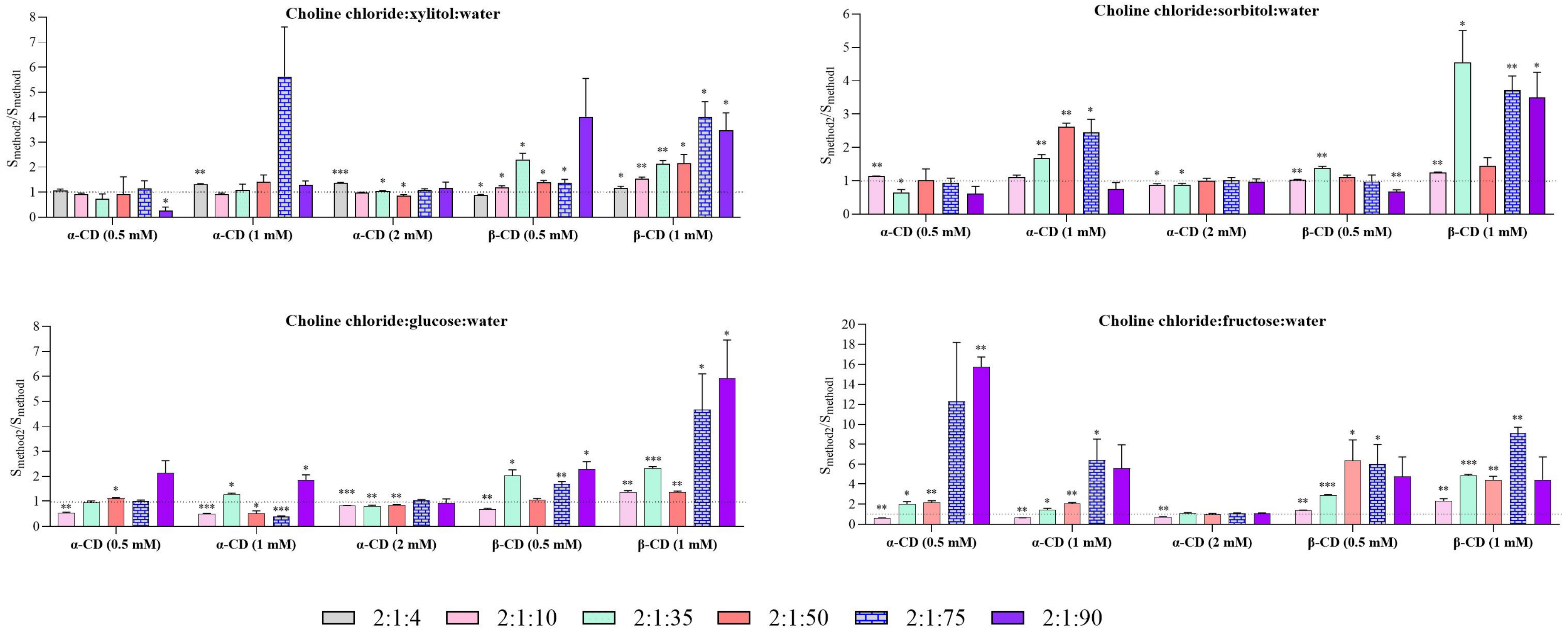

Figure S1. Solubility ratio of carvedilol obtained using Method 2 relative to Method 1 in DES and molar ratios through each cyclodextrins. Data are expressed as mean ± SD. A ratio value of 1 indicates equal solubility between both methods. Statistical significance compared to unity was assessed using a one-sample t-test (* $p < 0.05$, ** $p < 0.01$, *** $p < 0.001$).
